# Imaging and Spectroscopic-Based Methods to Understand Cancer Metabolism and Biology

**DOI:** 10.3390/metabo13080940

**Published:** 2023-08-12

**Authors:** Basetti Madhu

**Affiliations:** Cancer Research UK Cambridge Institute, University of Cambridge, Robinson Way, Cambridge CB2 0RE, UK; madhu.basetti@cruk.cam.ac.uk

## 1. Introduction

**Figure 1:**
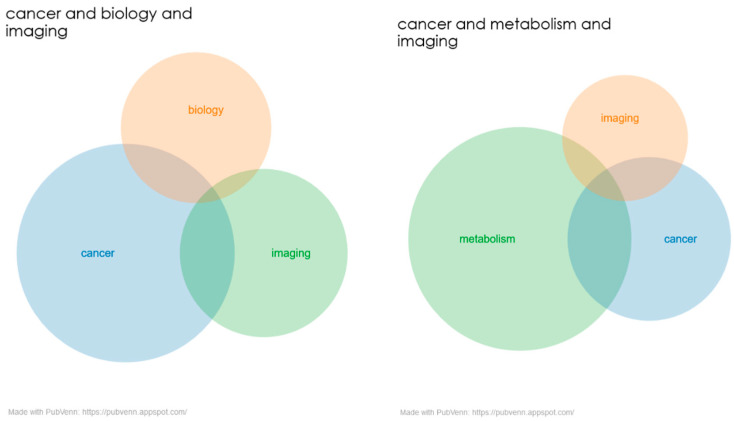
A Venn diagram of publications from PubMed.

The results of publications in PubMed with the MeSH terms “cancer”, “biology”, “imaging and cancer”, “metabolism” and “spectroscopy” are shown in Figure 1 in the form of a Venn diagram. These figures show small overlapping areas or intersections of the publications within these fields. This is one of the reasons to explore the imaging and spectroscopic applications in cancer biology and metabolism in the form of this Special Issue. These studies will expand our understanding of cancer biology and metabolism, which in turn can be applied in clinical routines for early detection and diagnosis, as well as monitor the progress and observe early responses to therapy and prognosis.

## 2. Cancer Biology

After the discovery of the DNA double helix structure in the 1950s, there was an enormous amount of research interest on the molecular biology of malignant tumours to better understand cancer biology and metabolism, which resulted in the subsequent wealth of biological data from cancer cells/tissues as well as the identification of several metabolic features of tumours. Many of these complex characteristics were organised into a framework of six biological capabilities by Hanahan and Weinberg in the year 2000. They included the features of a cell, which include sustaining proliferative signalling, evading growth suppressors, resisting cell death, enabling replicative immortality, inducing angiogenesis and activating invasion and metastasis as the hallmarks of the cancer. This scientific view has established that cancer is a complex disease involving dynamic changes in the genome. The publication of the human genome in year 2000 and its subsequent development of sequencing technologies hastened the accumulation of more molecular biology data and new biological features of cancers. Almost a decade later, in the year 2011, two emerging hallmarks were added to the existing original list of cancer hallmarks, namely the reprogramming of energy metabolism and the evasion of immune destruction. In addition, genome instability and mutation as well as tumour-promoting inflammation were suggested to be enabling characteristics. In a recent review article published in 2022, Hanahan further suggested that the cellular plasticity, non-mutational epigenetic reprogramming, polymorphic variations in organ/tissue microbiomes and senescent cells can also be considered hallmarks of cancer. At present, all of the aforementioned fourteen biological capabilities can be regarded as hallmarks of cancer. However, our knowledge of cancer biology remains lacking; it is therefore hoped that ongoing and future cancer research studies can shed more light on these aspects.

## 3. Cancer Metabolism

It is a well-known fact amongst biochemists working in the field of cancer metabolism that the excess lactate production in tumour slices, even in well-oxygenated conditions, was observed by Warburg in the 1920s. A higher consumption of both glucose (as a carbon source) and glutamine (as a nitrogen source) is needed to meet the demands of the growth and proliferation of cancer cells. Lipids and amino acid sources are also required by the cancer cells to meet the demands of the proliferation/growth of the cancer by synthesizing macromolecules, proteins, membranes and other cellular structural requirements. The metabolic adjustments that occur due to this excessive demand of nutrients, along with the dependence on the supply or availability of these ingredients in the tumour microenvironment for the growth of the tumour is now popularly known as metabolic reprogramming. Similar to biological capabilities organised into hallmarks of cancer biology, Pavlova and Thompson in the year 2016 proposed known cancer-associated metabolic changes into the following six hallmarks for cancer metabolism: (1) the deregulated uptake of glucose and amino acids, (2) the use of opportunistic modes of nutrient acquisition, (3) the use of glycolysis/TCA cycle intermediates for biosynthesis and NADPH production, (4) an increased demand for nitrogen, (5) alterations in metabolite-driven gene regulation and (6) metabolic interactions with the microenvironment. Spectroscopic methods such as NMR, mass spectroscopy, optical, infrared, photo-acoustic and Raman spectroscopy methods are used to study metabolism in cancer cells and tissues.

## 4. Imaging and Spectroscopic Methods

**Figure 2:**
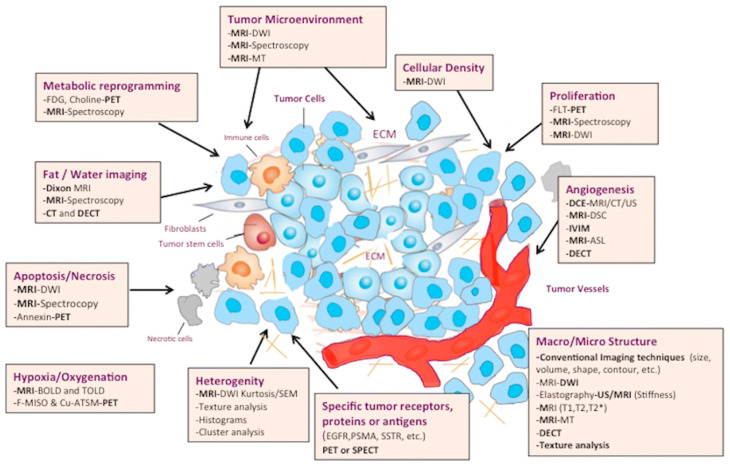
Main imaging and spectroscopic techniques in the evaluation of cancer biology, tumour metabolism and microenvironments (Figure 1 was reproduced with the permission from the article by García-Figueiras, R., Baleato-González, S., Padhani, A.R. et al., How clinical imaging can assess cancer biology. *Insights Imaging* **10**, 28 (2019). https://doi.org/10.1186/s13244-019-0703-0, https://insightsimaging.springeropen.com/articles/10.1186/s13244-019-0703-0#rightslink, accessed on 4 August 2023, is licensed under the Creative Commons Attribution 4.0 International License). Imaging methods shown in the figure are dynamic contrast-enhanced MRI (DCE-MRI), DCE ultrasound (US), dynamic susceptibility contrast-enhanced MRI (DSC-MRI), perfusion CT (PCT), diffusion-weighted imaging (DWI), magnetic resonance spectroscopy (MRS), spectroscopic imaging (MRSI), arterial spin-labelling (ASL), blood oxygenation level-dependent MR imaging (BOLD-MRI), elastography, positron emission tomography (PET) and single-photon emission computed tomography (SPECT) imaging. The picture also depicts the association of imaging and spectroscopic methods with several hallmarks of cancer biology from Hanahan’s article (2022) and cancer metabolism from Pavlova and Thompsons’ article (2016).

The spatial and structural information of internal body tissues can be obtained by various imaging modalities, whereas spectroscopy methods are commonly used to probe their functional biochemistry and metabolism. Multiscale imaging and spectroscopy modalities span the whole of the electromagnetic spectrum (see Table 1 for more details): Gamma rays (PET and SPECT), X-rays (X-ray and CT), the ultraviolet and optical region (bioluminescence, fluorescence and opto-acoustic imaging), infrared (thermal imaging, Raman imaging and spectroscopy), micro-waves (ESR/EPR) and radio-waves (NMR spectroscopy and MRI). Hyperpolarised ^13^C MRI and PET methods are used to study certain metabolic pathways in tissues with respective tracers. For example, FDG-PET images are based on glycolytic pathway functions, while FLT-PET shows cell proliferation. DCE-MRI methods are useful in investigating the function of tumour vasculature. Several imaging modalities can probe the heterogeneity of tumours and their microenvironments (see Figure 2). Deuterium metabolic imaging (DMI) has strong potential to become a dominant MR research tool and a viable clinical imaging modality to map metabolism with high temporal and/or spatial resolution in oncology.

The present Special Issue contains four reviews [1,2,3,4] and four research articles [5,6,7,8] with the original work covering a wide range of methods that can be applied to assess and measure several characteristics of tumours.

Prof Arús and Dr Candiota reviewed the status of imaging biomarkers for assessing the efficacy of immune system participation in glioblastoma therapy response and addressed the questions as to the means with which to produce them [1]. However, such biomarkers are not yet available, and current follow-up approaches for patients with glioblastoma are mostly based on MR imaging criteria or other approaches such as PET, which may overlook the information related to immune system oscillations. The paper suggests that adjusting follow-up protocols would be of great help to patients and health systems, saving time and resources instead of maintaining ineffective therapeutic strategies with few or no effects on patient survival or quality of life. The proposal is not confined to glioblastoma (GB) treatment with “classic” immunotherapeutic approaches, since most of the GB-efficient therapies are able to elicit a host immune response. The paper has limitations that need to be considered. The proposal is mostly based on results obtained with a single preclinical immunocompetent model (GL261), and with a limited set of therapeutic protocols. Additionally, the GL261 preclinical model does not fully emulate the whole human mutation panel, which could impact the observed MRSI spectral pattern. The paper also acknowledges that MRSI is not being routinely used in the clinical pipeline, either for diagnosis or for follow-up strategies, except in very specific circumstances. Moreover, MRSI acquisition is more challenging than MRI acquisition, and the observed metabolite signals have a lower concentration (and, hence, lower SNR) compared to water, which poses challenges in spectra acquisition and interpretation.

Prof Jagannathan and Dr Sharma’s review focuses on the applications of NMR, MRS and MRI methods in understanding breast cancer biology and its diagnosis and therapeutic monitoring [2]. The review discusses the potential of functional magnetic resonance (MR) methods in illustrating physiological and molecular process changes before anatomical manifestations on conventional MR imaging. In vivo proton (^1^H) MR spectroscopy (MRS) is widely used for differentiating breast malignancy from benign diseases by measuring elevated choline-containing compounds. The use of hyperpolarized ^13^C and ^31^P MRS enhanced the understanding of glucose and phospholipid metabolism. The metabolic profiling of an array of biological specimens can also be investigated through in vitro high-resolution NMR spectroscopy and ex vivo high-resolution magic angle spinning (HRMAS) NMR spectroscopy. The analysis of many NMR spectral datasets through multivariate statistical methods have classified the tumour sub-types. Multiparametric MRI approaches were found to be helpful in elucidating the pathophysiology of cancer by quantifying structural, vasculature, diffusion, perfusion and metabolic abnormalities in vivo. The paper concludes that these techniques have enormous potential in the development of new therapeutic approaches.

Dr Han et al. reviewed the status of photoacoustic imaging in biomedical research and concluded that the photoacoustic imaging has great potential to be used as a complementary modality to improve diagnostic accuracy for suspicious tumours in the thyroid and breast [3]. The paper also summarizes the methods and results of clinical photoacoustic trials available in the literature to date to classify cancerous tissues. The linear unmixing technique is commonly used for multispectral photoacoustic data analysis, but model-based unmixing and deep-learning techniques have also been used. The paper suggests that by combining structural information obtained by ultrasound imaging with molecular and functional information obtained by multispectral analysis, photoacoustic imaging can distinguish between benign and malignant tumours in vivo. The paper suggests that there are some limitations that need to be overcome for the successful clinical translation of multispectral photoacoustic analysis of cancer.

Dr Takakusagi et al. reviewed the use of Electron Paramagnetic Resonance Imaging (EPRI) and related magnetic resonance imaging techniques for mapping physiologic and metabolic aspects in vivo, with a focus on their application in cancer research [4]. The authors highlight the potential and challenges of each technique, including EPRI’s ability to provide quantitative images of tissue oxygenation, acidosis, redox and glutathione status. The paper also discusses the development of novel, narrow, single resonance, stable paramagnetic agents as tumour microenvironment probes, as well as the use of dissolution DNP (dDNP) for metabolic imaging. The authors note that while these techniques have not yet been widely used in clinical settings, they hold promise for future clinical applications.

Dr Deutsch et al. presented a non-invasive method to simultaneously measure exogenous reporters of fatty acid uptake and metabolic membrane potential and endogenous contrast related to haemoglobin concentration and tissue oxygenation [5]. The authors demonstrated the feasibility of this method by showing that Bodipy FL C16 (lipid phosphorylation surrogate) and TMRE (oxidative phosphorylation surrogate) do not crosstalk chemically, optically or biologically, and that these probes are compatible with an inverse Monte Carlo algorithm to extract tissue optical properties and intrinsic fluorescent signals. In vivo optical spectroscopy measurements of these simultaneously injected fluorophores revealed decreases in both TMRE uptake and total haemoglobin concentration in the mammary fat pads of healthy mice over the course of fifteen weeks. The authors observed that combinations of metabolic and vascular endpoints effectively delineated clusters of normal and tumour tissues where individual endpoints could not. These observations underscore the importance of examining the relationship between the metabolic and vascular variables instead of considering individual endpoints in isolation. The paper has broad implications in tracking metabolism in vivo to study disease progression and therapy resistance. The study was conducted on preclinical models, and the results may not be directly applicable to humans. The sample size of the study was relatively small, and further studies with larger sample sizes are needed to validate the findings.

Dr Alhulail et al. presented their 2D density-weighted concentric ring trajectory MRSI method as a reliable and practical non-invasive technique to quantify and map renal fat fractions [6]. It can be used to evaluate various renal diseases, such as diabetic kidney and renal tumours with their subtypes. The method has a high degree of repeatability and can be used within a clinically acceptable scan time at 3T.

Dr Agudelo et al. presented their patient-derived xenografts (PDXs) as realistic models of clear cell renal cell carcinoma (ccRCC) for imaging and metabolic studies [7]. However, the plasticity of metabolism must be considered when manipulating PDXs for preclinical studies. The metabolic comparisons of the PDX are congruent with the genetic clustering observed among them. The genetic stability observed between passages or upon the establishment of xenografts from cultured cells does not carry over to the metabolomic or imaging features, highlighting the plasticity of the metabolic phenotype and its dependency on microenvironment and growth conditions. These results emphasize the need to understand the irreversible streamlining of metabolic changes conferred upon the in vitro culture of cells from PDXs and the optimal choice of model systems for translational research in RCC, including drug screening and biomarker discovery targeting metabolic pathways.

Tumours are grown mostly in acidic microenvironments and Dr Irrera et al. investigated the effect of proton pump inhibitors (PPIs) on tumour acidosis in a preclinical model of prostate cancer using MRI-CEST tumour pH imaging [8]. The in vitro results showed that DU145 prostate tumour cells were sensitive to PPIs, with marked cell toxicity both in normoxia and in hypoxia, with hardly any change in pH. The in vivo studies were performed upon the administration of Esomeprazole to assess both the acute and chronic effects, and Iopamidol-based tumour pH imaging was performed to evaluate tumour acidosis. Although statistically significant tumour pH changes were observed a few hours after Esomeprazole administration, in both the acute study and up to one week of treatment in the chronic study, longer treatment resulted in a lack of changes in tumour acidosis. This was associated with similar tumour growth curves between the treated and control groups in both the subcutaneous and orthotopic models. Overall, the study highlights MRI-CEST tumour pH imaging as a valid approach to monitoring the treatment response to PPIs.

## Figures and Tables

**Table 1:** Electro-magnetic spectral ranges, underlying physical processes and ionization associated with the common imaging modalities.

Electromagnetic Waves	Physical Process	Frequency (Hz)	Imaging Modality		Ionization	Sound Waves
Gamma rays	Nuclear	10^26^			Ionizing	
		10^23^				
		10^19^	**SPECT**	**PET**		
X-rays	Core electrons	10^17^	**X-rays**	**CT**		
UV	Valance electrons	10^16^				
Visible		10^14^	**Optical**	**Fluorescence**	Non-Ionizing	
IR	Vibration	10^13^	**Raman**	**IR**		
Micro-waves	Rotation or electron spin transitions	10^10^	**EPR**			
		10^9^				
Radio waves	Nuclear spin transitions	10^8^	**MRI**			
		10^4^		**Ultrasound**		
Sound waves	Particles’ vibration in the medium	10^4^				Audible range
		10^2^				
		10^0^				
